# Analysis of the Behavioral Performance and Social Support of Patients in Online Health Communities From User Profile Perspectives: Comparative Study

**DOI:** 10.2196/68074

**Published:** 2025-09-23

**Authors:** Jie Wei

**Affiliations:** 1 Zhejiang University of Water Resources and Electric Power Hangzhou China

**Keywords:** online health community, user profile, social support behavior, text mining, social network analysis, k-means clusters

## Abstract

**Background:**

With the development of online health care, an increasing number of patients are consulting and exchanging social support through online health communities. People with different diseases have varying needs for information and emotional support. However, comparisons of similarities and differences in behavioral patterns among patients with different disease types and their social support needs require further exploration.

**Objective:**

Using a large-scale dataset of user-generated posts, we aimed to systematically examine how disease type (acute vs chronic) influences the behavioral patterns, emotional expressions, and support-seeking needs of users in online health communities, providing actionable insights for tailored community interventions.

**Methods:**

We identified patients with acute diseases and those with chronic diseases and then crawled corresponding user profiles and post data from the chronic disease online health community (CDOHC) and acute disease online health community (ADOHC). Using a pretrained model, we classified and described the social support performance of users. Subsequently, we conducted a comparative analysis of user behaviors, emotions, and needs by mining behavior patterns and textual content from posts. We performed further social network analysis using user profiles.

**Results:**

We identified 492,495 posts from 53,245 users in the CDOHC and 52,047 posts from 23,659 users in the ADOHC. Seeking and providing emotional support were higher in the CDOHC (83,231/492,495, 16.9% and 101,453/492,495, 20.6%, respectively), while seeking and providing information support were higher in the ADOHC (33,993/492,495, 22.8% and 61,128/492,495, 41.0%, respectively). These findings indicate that users with chronic diseases have a higher need for emotional support, while most users with acute diseases want to seek information support. The word co-occurrence network revealed distinct thematic patterns between the 2 communities. In the CDOHC, disease management clusters (8/17, 47%) and emotional clusters (7/17, 41%) showed balanced proportions, reflecting the dual needs of patients with chronic diseases. In contrast, in the ADOHC, posts were overwhelmingly focused on treatment (25/28, 89%), with minimal emotional vocabulary clusters (2/28, 7%). Social network analysis further highlighted these differences. The CDOHC showed the highest edge density in the seeking emotional support subnetwork and reciprocal interactions in 68.0% (83,025/122,095) of providing emotional support connections, indicating robust emotional support exchanges. Meanwhile, the ADOHC exhibited significantly faster post velocity in treatment discussions, consistent with its acute care context. These structural differences aligned with user behavior patterns. Users with chronic diseases maintained strong community bonds (averaging 8.2 connections/user), while users with acute diseases prioritized time-sensitive information (12,823/13,938, 92.0% of queries were related to treatment).

**Conclusions:**

This study contributes to a comprehensive understanding of how disease type influences the social behaviors and emotional expressions of users. The findings provide practical implications for doctors, patients’ families, and health care participants regarding targeted support strategies for patients with acute and chronic diseases.

## Introduction

### Background

Online health community (OHC) refers to an online community of individuals who use the internet to share or exchange knowledge on issues related to health or disease treatment [1]. The OHC eases the pressure of offline medical treatment and provides a platform for patients and their families to exchange information and share resources, enabling them to seek or provide social support. Social support refers to the resource exchange behavior of individuals to help the recipient [2]. According to the characteristics of the exchanged resources, psychologists have identified different types of social support [3], including information support, emotional support, accompanying support, and substantive support [4]. Prior studies have found that the type of social support is associated with disease progression and is an indispensable force in disease treatment [5]. Patients now routinely engage with various social supports in OHCs, as these platforms offer diverse services for individuals with different diseases, fostering a sense of belonging among users.

Research has shown that user needs for social support in OHCs vary depending on community type and user characteristics. This variation can be attributed to the fact that different disease groups face different medical conditions and psychological states, which in turn influence their behavioral manifestations and support needs in OHCs [6]. For example, considering the complexity of the disease and the long-term course of treatment, people with chronic diseases, such as diabetes, often have a stronger need for information support in OHCs [7]. Most women in communities involving breast and other gynecological diseases are anxious and worried about their diseases, and they value emotional support in community communication [8]. Patients with lung, colorectal, and pancreatic cancers have used OHCs mainly to meet their information needs, while patients with breast, ovarian, prostate, and skin cancers are most in need of emotional support [9].

Therefore, it is vital to investigate behavior differences across disease groups and tailor targeted support for diverse patient needs. However, while existing research in the literature has focused on analyzing the need for social support among patients within particular disease contexts, a comparative analysis to examine how these needs differ between disease types requires further exploration. Only by understanding the specific perceptions and needs of more patients will we be able to optimize the design of OHCs effectively. Beyond platform-level improvements, this knowledge would also enable policy makers and societal stakeholders to better grasp the macro-level characteristics of different disease populations, thereby providing them with more substantive social support.

To implement this comparative methodology, we concentrated on a basic distinction in disease classification: acute disease versus chronic disease. We have provided an additional literature review for pertinent concepts in the subsequent section.

### Acute Diseases and Chronic Diseases

According to the Merriam-Webster Medical Dictionary, an acute disease is characterized by rapid onset and relatively short duration, exemplified by conditions such as bronchitis, gastroenteritis, or influenza [10]. In contrast, a chronic disease, including asthma, coronary heart disease, and diabetes, persists or recurs over an extended period, representing a medical condition of prolonged duration [11]. The World Health Organization (WHO) also specifies chronic diseases as conditions that (1) persist for at least 1 year, (2) require ongoing medical management, or (3) limit activities of daily living. In this study, we have taken acute diseases and chronic diseases as classification cases to explore the differentiated impact of disease type on patients’ social behaviors in OHCs based on 3 key considerations. First, the divergent health care needs between these disease types shape distinct interaction patterns. Acute diseases (eg, infections and injuries) demand urgent, short-term support, leading to transient but intense OHC engagements [12], while chronic conditions (eg, diabetes and hypertension) foster long-term participation that cultivates stable peer-support networks for divergent health care needs [13]. Second, drawing upon the illness trajectory theory [14], we observe how fundamental differences in disease progression directly influence psychological states and help-seeking behaviors [15]. Acute conditions typically generate crisis-oriented interactions, whereas chronic illnesses promote ongoing relationship building in digital spaces [16]. Third, empirical research documents contrast digital engagement patterns. Acute patients demonstrate episodic platform use correlated with symptom exacerbations [6,17], while chronic patients maintain sustained participation and often occupy more central positions within support networks [18,19]. There can be differences in the characteristics of the disease itself, and acute and chronic diseases differ in their treatment objectives and strategies. The treatment of acute diseases is usually aimed at achieving a complete cure, while chronic diseases are difficult to cure. This classification approach provides critical insights for designing tailored OHC interventions that address the unique behavioral patterns emerging from these clinically distinct disease categories.

### Social Support

The conceptual evolution of the social support theory has undergone significant refinement since Durkheim’s work [20] in the late 19th century, building on foundational theories like the needs-fulfillment framework [21]. Contemporary research has adapted these concepts to digital contexts. Our study advances this theoretical progression by reconceptualizing OHCs as dynamic support networks, where social support emerges through the process of information and emotion exchange among users using postings and comments. Specifically, this study focuses on 3 types of social support: information support, emotional support, and companionship support. Information support entails offering information, advice, or guidance to community users, whereas emotional support pertains to the substance of pertinent emotional disclosure [22], including trust, caring, sympathy, etc. Such assistance can help patients reduce the stress or anxiety produced by health issues [23]. The concept of companionship support is comparatively broader, characterized by nonmedical interactions that foster community belonging.

Users may have different reasons and goals for using OHCs, and obtaining social support has become one of their key needs. The specific type of social support needed may be related to factors such as user characteristics, disease type, and psychological state. Existing research has explored the role of social support in both acute and chronic diseases. For example, the absence of a social support network significantly increases mortality risk following acute myocardial infarction [24]. Young and middle-aged individuals with weak social networks exhibit higher incidence rates of nonspecific chest complaints [25]. Moreover, rheumatoid arthritis patients with lower perceived social support demonstrate poorer emotional adjustment [26]. OHCs further amplify these effects. They serve as critical platforms for patient education and health communication [27]. Wang et al [28] analyzed and predicted user participation in OHCs from the social support perspective. They used text mining methods to decide what kind of social support each post contained. For chronic conditions, information support enhances self-care, while emotional support improves psychological health [29]. Network-based support is particularly beneficial for patients in managing asthma, diabetes, cancer, and rheumatoid arthritis [30]. Notably, longitudinal studies have confirmed that social isolation independently correlates with cardiovascular disease and type 2 diabetes progression [31]. People with neuroinflammatory diseases who received more social support during the COVID-19 pandemic were reported to have less mental isolation and better disease remission [32]. Women with chronic lipedema disease experience more stress when meeting with other people, and thus, they are more in need of emotional support [33].

### Computational Analysis Regarding OHCs

Recent research has leveraged computational methods to analyze social support in OHCs, employing techniques such as text mining, machine learning, and network analysis. For example, Wang et al [28] used machine learning to mine social support types from user posts, while Wu et al [34] applied latent Dirichlet allocation (LDA) topic modeling, albeit with manual feature extraction. Expanding these methods, Zhang et al [35] introduced a hybrid bidirectional long short-term memory–convolutional neural network model to detect information processing behaviors and paired it with LDA topic modeling to uncover distinct user needs. Linguistic analyses have further enriched this field. Jiang et al [36] quantified how linguistic features in Chinese OHCs correlate with support provision, and Gu et al [37] developed a natural language processing (NLP)-based cognitive change lexicon to assess psychological outcomes after support. Additionally, clustering techniques have revealed behavioral segmentation. Lu et al [38] identified 5 health-related topics and 3 stakeholder groups through expectation maximization, illustrating how users engage with health information. Network analysis has also emerged as a key methodology. For instance, Liu et al [9] adopted a network-based approach, overcoming prior limitations by analyzing word-level connections to reveal differences in social support needs across cancer types, whereas Lu et al [39] and Yang et al [40] adopted exponential random graph models to investigate structural patterns in informational and emotional support networks. Lin et al [41] combined social network analysis with word co-occurrence networks to study information-sharing behaviors among different user identities in an online tumor community. These studies demonstrate the value of text mining and network analysis in decoding the complexities of OHCs, offering scalable tools to examine support mechanisms, user roles, and community dynamics.

However, existing research usually fails to comparatively analyze how social support behaviors and needs differ between acute and chronic disease populations, particularly in the context of user profile research. While prior work has identified general patterns of support-seeking, critical disease-specific distinctions, such as the transient versus sustained support demands of acute versus chronic conditions, remain underexplored. This oversight limits the development of tailored interventions that account for the dynamic, context-dependent nature of social support in diverse disease trajectories.

### Study Aims

Our objective is to comparatively analyze differences in the characteristics of users with different disease types (acute and chronic) and their social support needs based on a large number of postings in OHCs. After categorizing the diseases into acute and chronic types, we employed multiple approaches to examine the combinations of emotions, behaviors, and user social support needs. This multi-method analysis helps enrich user profiles and deepen the understanding of the types of social support embedded in OHCs. To achieve this, we used user profile descriptions to characterize different user groups, including their behaviors and emotions, and applied deep learning models and social network analysis to examine the social support expressed in user postings. This allowed us to compare and analyze the behaviors, emotions, and needs of the 2 major user groups in OHCs, namely, users with acute diseases and those with chronic diseases.

While the analytical methods used are derived from existing literature, this study makes significant contributions to the field of OHCs by developing an integrated approach that combines user profiling, social support segmentation, and network analysis to better understand patient interactions and support dynamics. Methodologically, the research establishes a novel framework for comprehensive user profiling across 3 key dimensions, including information, behavior, and emotion, to enable more precise support delivery. By employing a hybrid approach that combines stratified sampling, manual annotation, and machine learning techniques, we successfully automated the classification of different social support types while maintaining high accuracy. This approach revealed differences in support-seeking behaviors across different disease communities. Furthermore, the construction of multi-layered social networks provided valuable insights into community engagement patterns and helped visualize how users exchange different types of support. From a practical perspective, these findings offer concrete benefits for community management by helping moderators identify the needs of different users and optimize support allocation. The research also informs patient-centric design and demonstrates how OHCs can effectively supplement traditional health care by fostering peer-support networks.

## Methods

### Data Sources

In this study, we used crawler technology to capture the public data of chronic and acute diseases in China’s representative OHCs, such as Haodf, Baidu Medical Forum, and “Sweet Homeland” community. We chose these online health care platforms for 3 key reasons. First, these OHCs are among the largest OHCs in China, featuring comprehensive functional modules, high consultation volumes, tens of thousands of active users, strong user retention, and broad coverage of diverse diseases. Second, the platforms cater to similar user groups, primarily patients with chronic or acute diseases (varying severity) and their family members. These users engage in mutual interactions for disease counseling and experience sharing. Third, by including multiple similar but nonidentical OHCs, we ensured sufficient data volume for the studied diseases while leveraging interplatform differences to enrich data indicators and construct a more complete user profile.

As illustrated previously, we have taken acute diseases and chronic diseases as classification cases to explore the differentiated impact of disease type on patients’ social behaviors in OHCs. According to the World Health Statistics Report issued by the WHO, cardiovascular diseases, cancer, diabetes, and chronic kidney disease are classified as chronic diseases. Meanwhile, influenza A infection, COVID-19, acute appendicitis, and cerebral hemorrhage are considered acute infectious diseases. Based on this classification, we selected cardiovascular diseases, cancer, diabetes, and chronic kidney disease to represent chronic diseases in this study, and they were part of the chronic disease online health community (CDOHC). Moreover, we selected influenza A infection, COVID-19, acute appendicitis, and cerebral hemorrhage to represent acute diseases, and they were part of the acute disease online health community (ADOHC).

In this study, we set the time window for data collection from January 2015 to April 2023, and crawled the user homepage information and user post data for corresponding diseases from the CDOHC and ADOHC. The homepage information included comprehensive demographic statistics, such as gender, birth date, geographical location, registration duration, and self-reported health status. The user post data encompassed all user-generated content, including discussion threads, comments, and timestamps of interactions. For example, the data crawled from the “Sweet Homeland” community forum were categorized according to the disease classification “type I, type II, and diabetes complications.” In the type II diabetes module, 80,120 original posts and 234,648 corresponding replies were crawled, totaling 314,768 posting records. In the type I diabetes module, 13,750 original posts and 48,059 corresponding replies were crawled, totaling 61,809 posting records. In the diabetes complications module, 2448 original posts and 11,740 corresponding replies were crawled, totaling 14,188 posting records. From the CDOHC, final discussion topics of 53,245 chronic disease users were crawled, and a total of 492,495 data records were collected. From the ADOHC, 52,047 raw posting data records from 23,659 acute disease users were crawled, and these were combined with their corresponding reply posts, totaling 149,095 records. The dataset covers tens of thousands of topics.

### Study 1: User Profile

The goal of this study is to analyze the performance of patient users and their social support, and thus, capturing user information is the first critical step. Building user profiles is an effective way to understand user needs, gain real-time insights into user preferences, achieve service transformation, and solve the problem of asymmetry between users’ precise needs and extensive services in OHCs [42]. At present, there is a lack of research on the application of user profile technology to OHCs. Based on the research objectives, static and dynamic tags were established for different users, and user characteristics were presented through tags [28]. Zhang et al [35] constructed a conceptual model of diabetes circle user profiles from the 3 dimensions of user needs, roles, and behaviors, and expressed different characteristics of users through group clustering [43]. User profiles can be realized with the following 3 steps: data mining, feature extraction, and portrait presentation. Among them, the construction of a user profile feature indicator system is important to realize user profiles. Accordingly, the index data of each user were obtained, and each dimension was categorized and counted. Finally, the user profile features in the community were presented by the clustering method.

**Figure 1:**
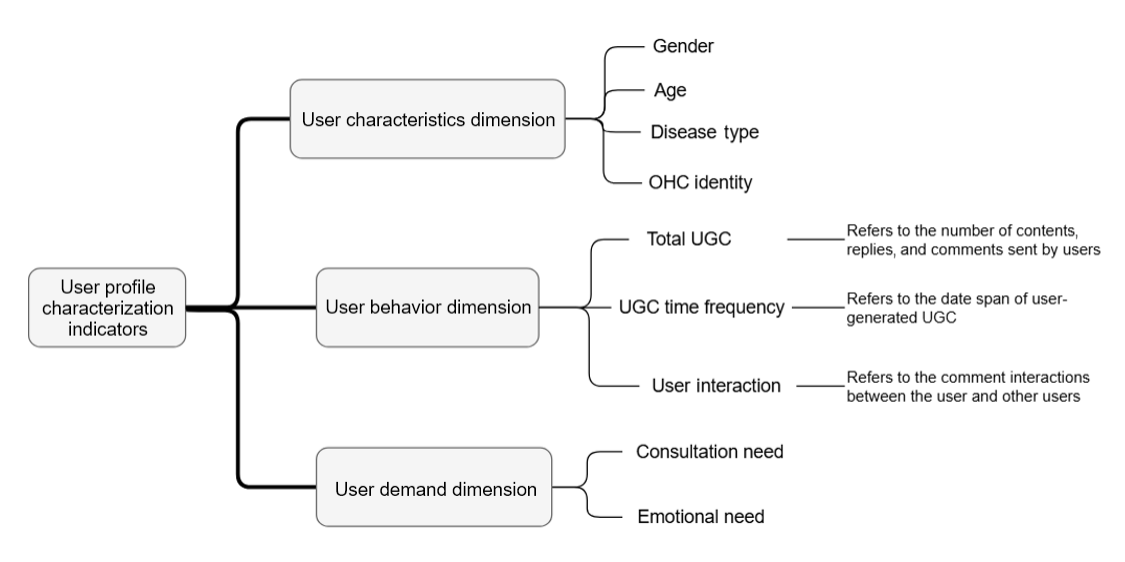
User profile feature system. OHC: online health community; UGC: user-generated content.

Observing the user information included in the 2 communities, the user profile feature system constructed in this study included the following 3 dimensions: user information (gender, age, disease, etc), user behavior (total amount of user-generated content [UGC], interaction, frequency of participation, etc), and user emotions (negative and positive). The specific indicator system is provided in Figure 1.

Indicators for the user information dimension can be obtained directly from the community without processing. The total number of UGCs in the user behavior dimension is the total number of posts, replies, and comments posted by users in the community, which can reflect the interaction of users in the community. The UGC time frequency indicates the date span of the user’s interaction and posting in the community, which can reflect the strength of the user’s stickiness to the community. The k-means analysis method has the advantages of high efficiency and scalability when clustering large datasets, which are in line with the characteristics of the large sample data in this study. The sentiment dimension is analyzed by text processing of the user’s UGC posting and sentiment analysis by algorithms. In Python, we used the Baidu Sentiment Analysis interface to recognize the sentiment tendency of all user posts crawled. User profiling relies heavily on label clustering, and there are many approaches to clustering, including density-based clustering, model-based approaches, and hierarchical and segmentation methods. In this study, we chose the k-means algorithm to cluster all the samples to obtain the number of user profiles.

### Study 2: Segmentation of Social Support Types

We categorized user-posted content (posts and comments) in terms of social support to identify the social support needs of patients with different diseases in different communities. There are various research approaches in existing studies to categorize the content posted by users of OHCs. In a prior study, posts were manually coded for 7 different social support categories, including achievements, congratulations, network support, seeking emotional support, seeking information support, providing emotional support, and providing information support [42]. The most salient social support categories at different stages of diabetes were also compared through a mixed research approach. Unlike traditional research that relies entirely on manual coding, another study introduced a text mining approach to automatically identify large-scale data for social support on the basis of a small amount of manually labeled data [44]. A classifier was trained based on machine learning techniques to determine which category or categories of social support each post contains [44]. In this study, we used the ERNIE2.0 model in machine learning to categorize the text of users’ postings, which was consistent with the definition of social support in previous studies. The categories included seeking information support, providing information support, seeking emotional support, providing emotional support, and companionship support. Since the content of accompanying support posted by users often involves participating in community activities or posting some daily topics, such as punching cards, recording one’s daily diet, or talking about the weather and traveling in the community, we did not classify the category of accompanying support as “seeking” and “providing.” Table 1 provides examples of social support for user-posted comments in the 2 communities (CDOHC and ADOHC).

**Table 1:** Example posts of social support.

Type	CDOHC^a^	ADOHC^b^
Seeking information support	“Do I need to inject insulin in my case?”	“My child has the flu and has a fever of 38 degrees in the middle of the night, what medicine should I take?”
Providing information support	“All pregnancies are treated with insulin, and the side effects of the medication can have an effect on the child.”	“Quadrivalent influenza vaccine is one of the preventive vaccines recommended by the World Health Organization, mainly used to prevent the four influenza viruses, quite effective.”
Seeking emotional support	“Can someone please comfort me? It feels like it's impossible to hold on!”	“The kids at home have been coughing and it hurts me so much to hear that.”
Providing emotional support	“Don’t be so pessimistic, eat right, exercise properly and stick with it and you’ll be fine!”	“Don’t be discouraged. We’ll beat this virus.”
Companionship support	“Day 8 of the diabetes diagnosis. Hang in there, family.”	“The number of covid-19 is increasing a lot day by day, so wear a good mask!”

^a^CDOHC: chronic disease online health community.

^b^ADOHC: acute disease online health community.

This module adopts the intelligent labeled data function provided by Baidu AI. The pretraining ERNIE2.0 model can predict unlabeled data after completing the learning of a small amount of manually labeled data, so as to obtain large-scale intelligent labeled data. The structure of ERNIE2.0 is the same as that of BERT. ERNIE2.0 is mainly for the modification of pretraining tasks to improve the effect. The continuous pretraining framework of ERNIE2.0 aims to extract lexical, syntactic, and semantic information from the training corpus, which incrementally builds pretraining tasks, and then, pretraining models learn on these built tasks through continuous multitask learning. After data preprocessing and screening, we extracted 3500 text records from the total text volume (about 550,000 text records) using random sampling for manual annotation. These texts were then annotated by 5 trained annotators, who categorized each post according to the predefined social support taxonomy. We performed a reliability check between multiple raters to ensure consistency in the manual labeling process. The interrater agreement was assessed by Fleiss kappa, and a score of 0.875 was achieved, suggesting good performance regarding interrater reliability. Additionally, the final labels were validated by 10-fold cross-validation. The annotation results indicated satisfactory reliability. Then, ERNIE2.0 was used to intelligently annotate the remaining unannotated text records. We split the labeled dataset into a 70% training set and a 30% test set to develop and evaluate our classification model. The proposed model finally achieved an annotation accuracy of 86.3% after comparing the performance of different algorithms for classification.

### Study 3: Social Support Network Analysis

#### Word Co-occurrence Network Construction

A co-word network is a network built from the perspective of the co-occurrence relationship between keywords to study the association and influence between different keywords [45]. The co-occurrence network was constructed through the following steps. First, we segmented the textual data of the posts crawled from the 2 communities. After performing word frequency analysis of the segmented terms, we merged the synonyms and removed low-frequency words. Subsequently, the top N keywords were selected to generate the word co-occurrence matrix. The keyword co-occurrence network was generated according to the keyword co-occurrence patterns. In this network, any 2 words were considered semantically related if they co-occurred in the same context. Nodes represented keywords, and edges represented co-occurrence relationships. The node size represented the word’s relevance magnitude, which corresponds to the number of co-occurring associations. Larger nodes indicated higher relevance and greater importance of the represented words. If a word was connected to many other words, it was located at the core of the network graph. Second, 2 words with co-occurring relationships in a network graph were connected by a line, which refers to how many raw data reports these 2 words appear in at the same time. This is usually indicated by the thickness of the connecting lines, but in this study, connecting lines of the same thickness were used for the overall effect.

#### Network Analysis of Social Support

In OHCs, users naturally form interconnected social networks where nodes represent individual users and edges capture the various types of social support exchanged through their interactions. The network construction began by analyzing user communications, including posts, comments, and replies, to identify and classify different support relationships. The types of social support were identified based on how and what users communicate with each other, thus connecting multiple users into a multi-relational social network, where each connection type reveals distinct patterns of community engagement. For example, when one user responds to another’s questions, this creates an information support link. Moreover, when users share personal experiences or daily routines, companionship bonds emerge. Furthermore, when users exchange comforting messages, emotions support connection formation.

**Figure 2:**
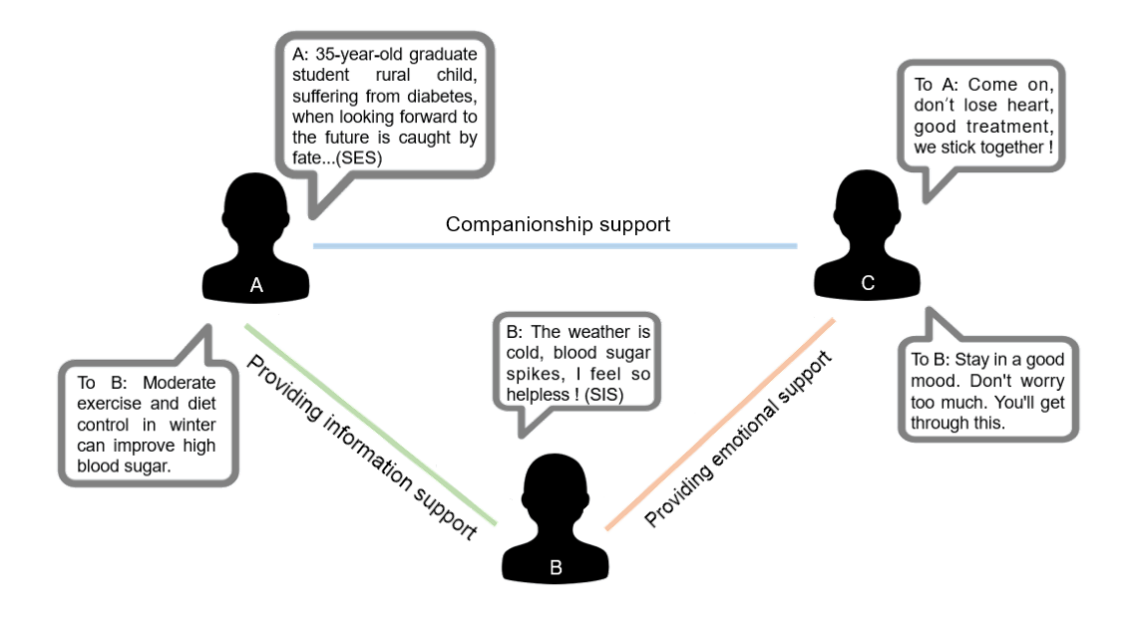
Display of social network relationships between users. SES: seeking emotional support; SIS: seeking information support.

To build this network systematically, we extracted interaction data from user posts, comments, replies, and mentions in the community forum. Based on user identification and text cleaning, we combined manual annotation and deep learning–based NLP to categorize social support types. Nodes represented users, and edges represented directed support relationships, where a connection from user A to B indicated that A provided support to B through posts or comments. The network adopted a multilayer structure, with distinct layers for different support types (informational, emotional, etc). The social network relationships between users are shown in Figure 2.

In Figure 2, user A posts a post expressing despair after experiencing diabetes, and the user seeks emotional comfort. User C responds to user A’s post and offers to work together to overcome the disease, providing companionship support for user A. Users A and C connect through companionship support and emotional support. User B posts a help post expressing that, as winter temperatures drop and blood sugar levels spike, there is a feeling of helplessness, and the user seeks support information. User A replies to user B about ways to relieve blood sugar elevation, and users A and B get connected through information support. User C pays attention to user B’s helplessness and provides comforting and encouraging emotional support. Users B and C are connected through this connection.

Different kinds of social support behaviors constituted separate subnetworks that formed an aggregated network by overlaying them. The number of nodes represented the number of users involved in a certain type of social support in the network. Connections represented certain social support behaviors between 2 users in a community. Density reflected the overall activity of the network. By statistically analyzing the social support network of users in OHCs, the user overview of user social support in online communities can be better presented, and the performance of user social support in communities with different disease types can be compared.

### Ethical Considerations

The study exclusively analyzed publicly available data from open online communities where users voluntarily share content with an expectation of public visibility. In strict adherence to established internet research ethics guidelines [46], we implemented comprehensive privacy protection measures, including complete anonymization of all data through removal of usernames, profile information, and any potentially identifiable metadata. Particular care was taken to avoid verbatim quotations that might enable search engine identification, and all analyses were conducted at an aggregate post level rather than an individual user level. The public nature of the source data and these protective measures were reviewed and determined to meet the exemption criteria by the Zhejiang University of Water Resources and Electric Power institutional ethics review board. Throughout the research process, we maintained vigilance regarding potential privacy implications to ensure no harm could come to any individuals whose public posts were included in our analysis.

## Results

### User Profile Results

The k-means method was used to implement user profiles for the 2 communities (CDOHC and ADOHC). To determine the optimal number of clusters (k) for k-means clustering, we applied the elbow method by computing the within-cluster sum of squares (WCSS) for k values ranging from 1 to 10. The WCSS curve typically exhibits a characteristic elbow point where the rate of decrease slows substantially, indicating the optimal k value that balances model complexity and clustering performance. This inflection point represents the point of diminishing returns for additional clusters. In the CDOHC, the clustering effect was optimal when k was taken as 3, while in the ADOHC, the clustering effect was optimal when k was taken as 5. The output clustering result was the user profile. The results are shown in Tables 2 and 3.

**Table 2:** User profile results of the chronic disease online health community.

Variable	Category 1	Category 2	Category 3
Disease type	Type I and II diabetes, and cancer	Type II diabetes, cancer, and chronic kidney disease	Cardiovascular disease, type II diabetes, and chronic kidney disease
OHC^a^ identity	Newcomer	Newcomer	Resident user
Interaction	Low	Middle	High
Total UGC^b^	Low	High	High
UGC time frequency	Low	High	High
Emotional tendency	Negative	Negative	Positive

^a^OHC: online health community.

^b^UGC: user-generated content.

**Table 3:** User profile results of the acute disease online health community.

Variable	Category 1	Category 2	Category 3	Category 4	Category 5
User gender	Woman	Woman	Man	Woman	Man
User age	Elderly patient	Young patient	Minor patient	Elderly patient	Elderly patient
Symptom	Suspected flu	Acute abdominalgia	Acute abdominalgia/flu	Suspected flu/COVID-19	Cerebral hemorrhage
Total UGC^a^	Low	Low	High	High	Low
UGC time frequency	Low	Low	High	High	Low
Demand dimension	Therapeutic consultation	Therapeutic consultation	Therapeutic consultation	Pathological knowledge	Therapeutic consultation

^a^UGC: user-generated content.

In the CDOHC, users were divided into 3 main categories. Category 1 was dominated by newcomers with type I and II diabetes, and cancer. Their overall participation in the community was low, and their postings were generally negatively inclined. Category 2 covered users with type II diabetes, cancer, and chronic kidney disease, who were also new users but had higher UGC output and community stickiness, and posted UGC text with a predominantly negative affective tendency. This was mainly related to newcomer status. Patients tended to have low psychological acceptance and a pessimistic view of the disease at the time of diagnosis of the disease. The chronic disease users in category 3 were resident users in the community, indicating that they had registered in the online community for a long time and logged into the community frequently. Moreover, the interaction rate, UGC output, and stickiness of this group of users were high, and the posts posted had positive emotions such as encouragement and persistence. From the results of user profiling in the CDOHC, the number of online registered groups of type II diabetes patients was high. The frequency of logging into the community and posting, and the stickiness of the community were moderate for most of the patients. A vast minority of users, as shown by the results in category 3, were active in the online community and were good at communicating positive emotions and support.

In the ADOHC, users were divided into 5 categories. Both category 1 and category 4 were dominated by older female patients. The difference was that category 4 had a portion of patients with COVID-19, and their UGC output was significantly higher than that of category 1. This was due to the fact that a novel virus has a higher degree of unknowns, and there was a greater need for people to obtain information about the risks of the disease and treatment options through the internet. Categories 2 and 3 had younger user groups, and category 2 was dominated by users with acute appendicitis. The overall low activity and participation of this segment of users in the OHC may be related to the fact that the onset of the disease is sudden and usually requires offline surgery. Category 3 mostly included underage patients, and although the gender of the users was mostly male, their family members were likely to be the ones who asked for treatment in the community. This group was highly active and sticky, which is in line with the popularity of online consultation. Category 5 included older male patients experiencing cerebral hemorrhage and other medical conditions. This group was generally older, and most patients were over 50 years old. Their needs in the OHC mainly involved medical treatment and consultation.

Comparing the results for the user profiles of the CDOHC and ADOHC, we found that there were more users with higher UGC output and stickiness in the CDOHC, which was dominated by chronic diseases, than in the ADOHC, which was dominated by acute diseases. This is related to the pathological characteristics of the 2 types of diseases. Chronic diseases have long disease cycles, with users going from diagnosis to near cure over several years. Users also communicate and ask for medical advice more frequently in online communities. While patients with acute illnesses usually choose offline emergency treatment at the first event of the acute illness, for diseases with mild and common symptoms or for situations that require health care after consultation, patients will choose to seek help from online communities. Therefore, the user base of the acute disease community is updated quickly, and the overall stickiness and continuous participation are relatively low.

### Social Support Results of Users

The results in Table 4 show that there are differences in users’ needs for social support and in their behaviors in both the CDOHC and ADOHC. In the CDOHC, the proportions of user posts seeking information support (84,709/492,495, 17.2%) and emotional support (83,231/492,495, 16.9%) were similar. On the other hand, the proportion of user posts providing information support (122,631/492,495, 24.9%) was slightly higher than the proportion providing emotional support (101,453/492,495, 20.6%). This suggests that the overall difference between users’ needs for informational and emotional support in the chronic disease community was not very large. There were many users who, in addition to counseling about their illnesses, also engaged in emotional exchanges between themselves and other users in the online community. For patients with chronic diseases, emotional support is a very important social support, which can help them relieve disease anxiety and achieve psychological comfort.

**Table 4:** Comparison of social support behaviors between different diseases.

Social support type	Results for CDOHC^a^ (N=492,495), n (%)	Results for ADOHC^b^ (N=149,095), n (%)
Seeking information support	84,709 (17.2)	33,993 (22.8)
Providing information support	122,631 (24.9)	61,128 (41.0)
Seeking emotional support	83,231 (16.9)	9392 (6.3)
Providing emotional support	101,453 (20.6)	13,866 (9.3)
Companionship support	61,561 (12.5)	5218 (3.5)

^a^CDOHC: chronic disease online health community.

^b^ADOHC: acute disease online health community.

In the ADOHC, the differences between different social supports were more significant. Users’ needs and the provision of information support were more prominent. Among them, the proportion of user posts seeking information support reached 22.8% (33,993/149,095), and the proportion of user posts providing information support was 41.0% (61,128/149,095). Regarding emotional support, the proportions of user posts seeking (9392/149,095, 6.3%) and providing (13,866/149,095, 9.3%) emotional support were both less than 10%. This suggests that in the acute community, users’ communication topics cover much more at the information level than at the emotional level, which indicates that the behaviors of patients with acute illnesses in online communities are more related to the expectation of disease-related information, and a small proportion of users are involved in communicating and supporting their emotions.

The longitudinal social support comparison between the CDOHC and ADOHC revealed that the overall needs and behaviors of users in the CDOHC were closer to each other in terms of information support and emotional support, while users in the ADOHC desired much more information support than emotional support. We then conducted a cross-sectional comparison of the 2 communities and found that even though the proportion of emotional support in the CDOHC was not as large as that of information support, this value was much larger than the proportion of emotional support in the ADOHC. Moreover, the difference between the 2 communities for companionship support was significant. The need for companionship support in the CDOHC (61,561/492,495, 12.5%) was significantly higher than that in the ADOHC (5218/149,095, 3.5%).

Combining the results of the previous 2 studies, the characteristic portraits of users in the 2 disease communities were fuller and the differences were more distinct. The user profiles mainly clustered the users in the CDOHC and ADOHC by different characteristics and extracted the behavioral trends and differences between the 2 types of disease patients. The performance of these users on social support was segmented, and the segmentation results reflected the differences in their needs for different social supports.

In the CDOHC, the behavioral characteristics of users were more similar overall, and there were fewer classified clusters. Users were more engaged and sticky in the OHC and demonstrated a higher need for emotional support and companionship support in their social support performance. This appears to be related to the psychological effects in patients with chronic diseases. User demand for OHCs was related to not only diagnosis and treatment counseling but also emotional communication and companionship with peers. This can lead to a higher reliance on OHCs and an expectation to receive appropriate support and feedback from them. In the ADOHC, users were divided into 5 different categories, with significant differences between categories and similar user behaviors within each category. Users within this community exhibited more pronounced differences in behavioral characteristics. Overall, patients with acute diseases and their families had a high demand for information support related to disease treatment, but they had a relatively low demand for emotional support and companionship support related to psychological comfort and caring encouragement. Users tended to end their behaviors after exchanging information in a short period of time, lacking emotional dependence, and the stickiness and continuity of user behaviors were weak.

Combining the results of user profiling and social support classification, we have 2 important findings. First, users’ behavioral signs in OHCs corresponded to their needs. Patients with chronic diseases who had higher emotional support needs were more dependent on OHCs. Second, users’ needs for social support were related to their disease types. For acute diseases with a fast onset and short cycle, users’ needs for timely and effective information support were much higher than their needs for emotional and daily companionship. To further understand the key topics of the post texts in the 2 types of OHCs and users’ engagement behaviors in each social support category, we conducted network analysis.

### Results of Network Analysis

#### Word Co-occurrence Network of the CDOHC and ADOHC

We conducted a word co-occurrence network analysis of the posts used to categorize social support in OHCs in study 2. As the total number of posts in the CDOHC and ADOHC reached over 10,000, the directly generated post co-occurrence networks contained too many words for effective visualization. We used randomly selected posts from the 2 types of communities, which were required to cover at least the postings of users in each type of clustered group. These postings were then subjected to word co-occurrence networks separately, which clustered words together without breaking their semantic links.

**Figure 3:**
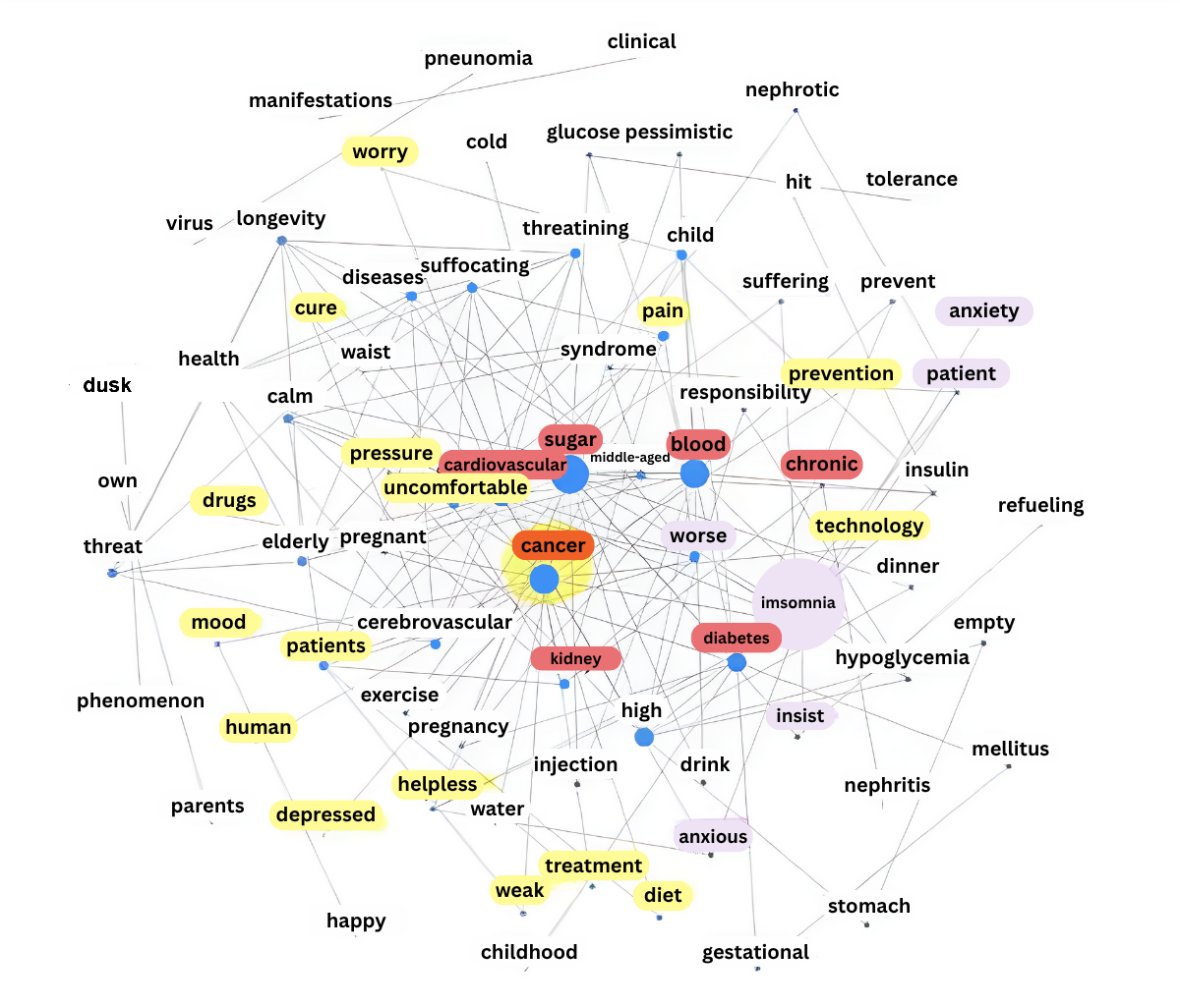
Word co-occurrence network in the chronic disease online health community.

Regarding the CDOHC, the center of the network in Figure 3 includes words related to chronic diseases, such as cancer, blood sugar, etc, which are represented by red. Topics related to diseases are mainly focused on the treatment and feelings of patients. The yellow area in Figure 3 indicates the selection of “cancer” as the keyword. It can be inferred that the topics in this module are related to information support and emotional support. The purple area in Figure 3 indicates the selection of “insomnia” as the keyword, and the related topics include words such as anxiety and helplessness. It can be inferred that the theme of this module is mainly related to the emotional communication of users. In addition, some topic words in Figure 3 are related to diseases, and they correspond to information support.

**Figure 4:**
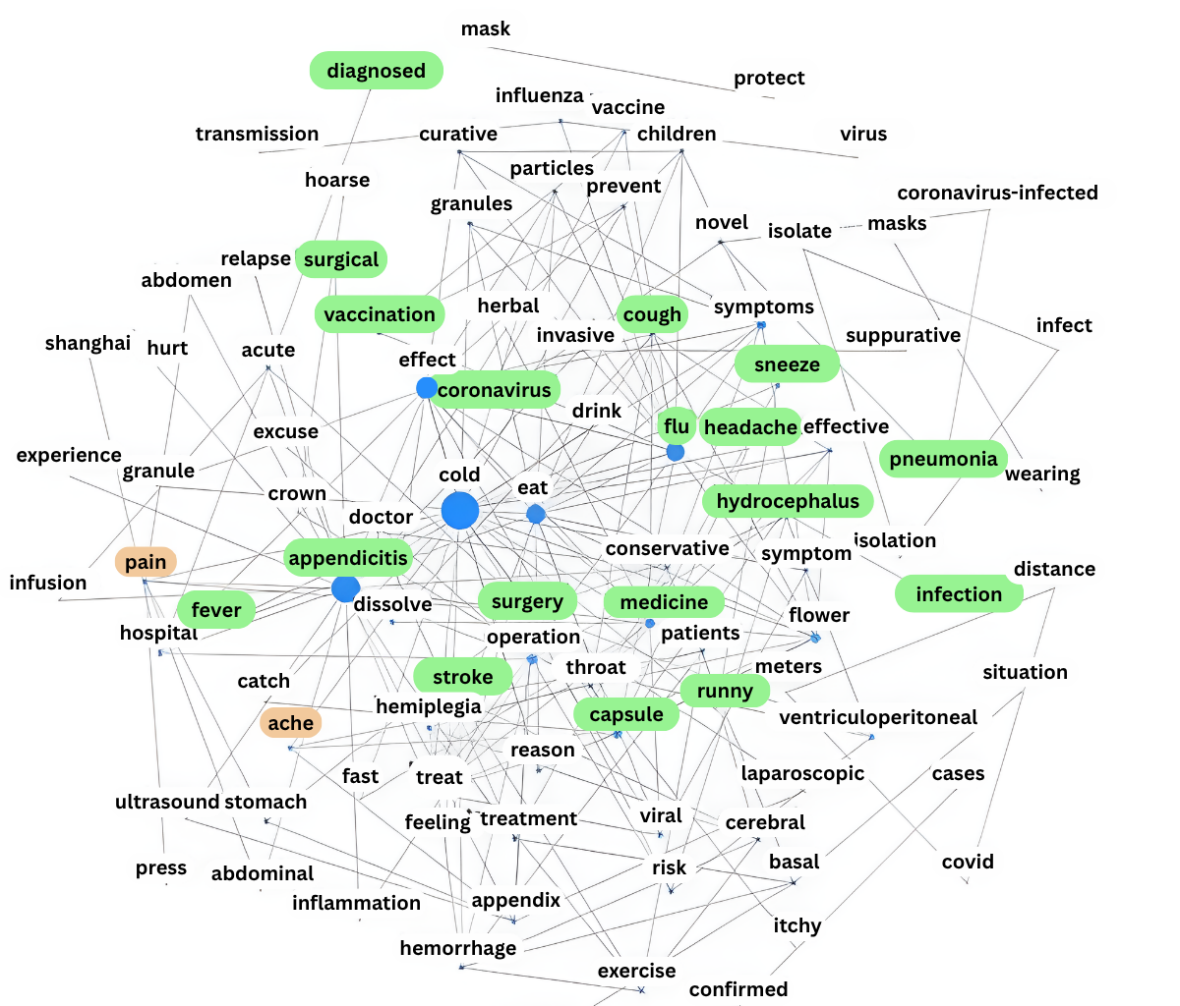
Word co-occurrence network in the acute disease online health community.

Regarding the ADOHC, in the network in Figure 4, the nodes of diseases, such as influenza and appendicitis, are larger. We found that most of the regional keywords in the network map can be categorized as treatment, symptoms, and drugs, such as the words in the green area in Figure 4. This shows that in acute posts, users mainly exchange information about treatment and medicine. Moreover, a small number of topics focused on words such as pain and fear.

Comparing the word co-occurrence network graphs, words in a module are likely to describe closely related themes. The 2 main modules in the network of the CDOHC can be seen as describing information support and emotional support. Most of the words in the network of the ADOHC appear to be related to disease treatments and medicine names and can be considered information support. A small number of OHC posts were selected for word co-occurrence network visualization to quantify the semantic structure of the posts, and the results of the analysis further validate the findings of the segmentation of social support.

#### Social Network of the CDOHC and ADOHC

We analyzed multiple social networks in OHCs for acute and chronic diseases. Tables 5 and 6 provide descriptive statistics for the aggregated network and 5 subnetworks. In the CDOHC, the seeking information support and providing emotional support subnetworks had the highest number of nodes and a close number of edges. The number of nodes in the providing information support and seeking emotional support subnetworks was close, but the number of edges and density of the seeking emotional support subnetwork was greater than that of the providing information support subnetwork. This suggests that in the CDOHC, there are more active users of both informational and emotional support, with users in the emotional support subnetwork being more closely connected to other users. The number of nodes and edges, and the density of seeking information support and providing information support were the largest among all subnetworks of the ADOHC. This indicates that users are most active on information support topics and post the most content. Comparing these 2 communities, user participation status varies in different subnetworks. The overall activity of user participation in the CDOHC was better, and users in this community were more connected to each other. The high activity and connection of users in the ADOHC were mainly reflected in information support, and all other subnetworks were more sparse.

**Table 5:** Descriptive statistics of the chronic disease online health community.

Property	Aggregated network	Subnetwork				
		SIS^a^	PIS^b^	SES^c^	PES^d^	COM^e^
Node, n	31,258	29,935	19,629	20,591	28,599	19,672
Connection, n	145,928	128,765	82,153	126,542	122,095	93,671
Density	0.05	0.04	0.03	0.05	0.05	0.04

^a^SIS: seeking information support.

^b^PIS: providing information support.

^c^SES: seeking emotional support.

^d^PES: providing emotional support.

^e^COM: companionship support.

**Table 6:** Descriptive statistics of the acute disease online health community.

Property	Aggregated network	Subnetwork				
		SIS^a^	PIS^b^	SES^c^	PES^d^	COM^e^
Node, n	16,927	13,663	13,258	10,801	10,263	9538
Connection, n	56,283	42,362	38,965	18,334	15,956	13,505
Density	0.03	0.03	0.02	0.004	0.003	0.001

^a^SIS: seeking information support.

^b^PIS: providing information support.

^c^SES: seeking emotional support.

^d^PES: providing emotional support.

^e^COM: companionship support.

### Summary of the Findings

**Figure 5:**
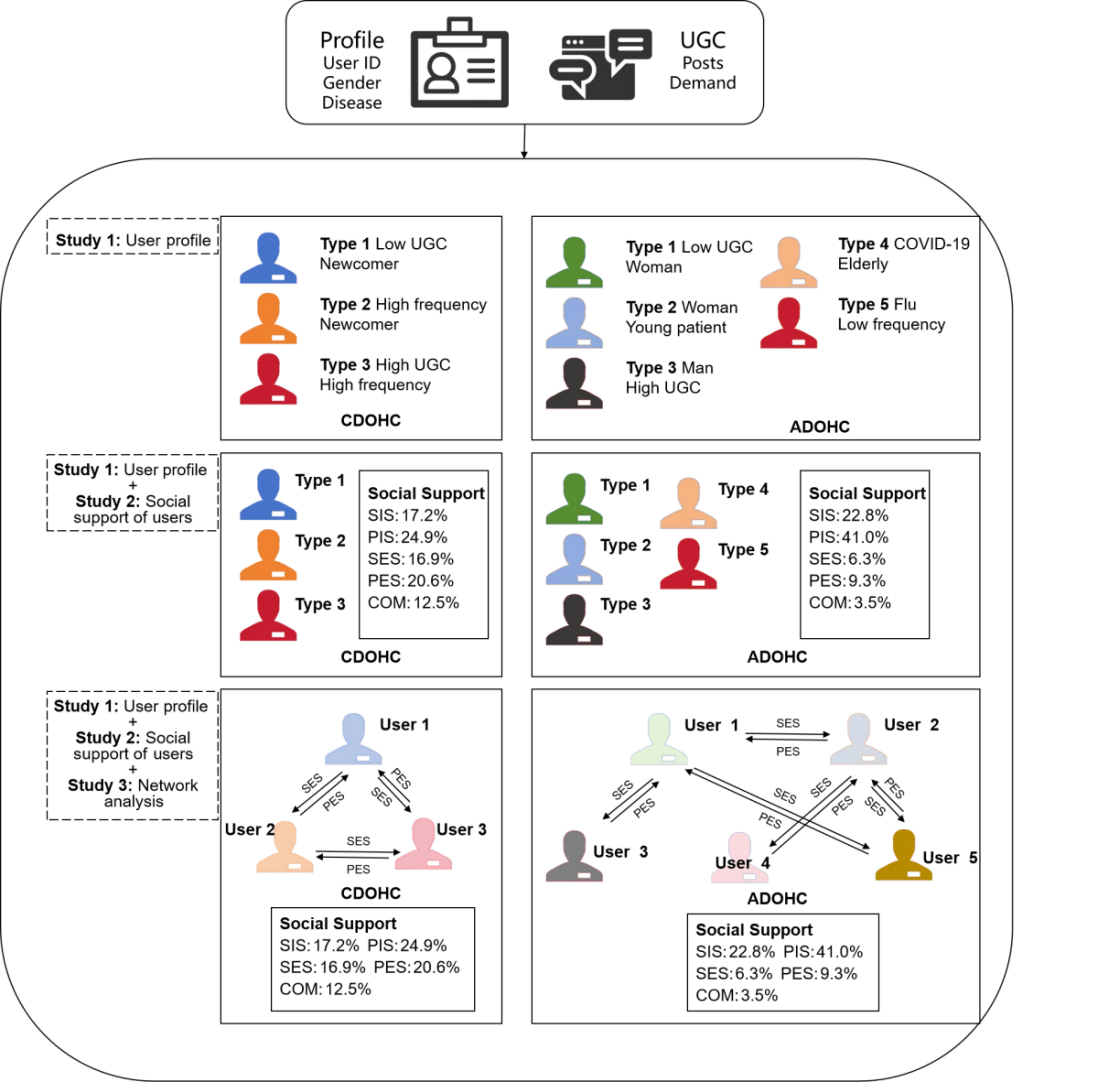
Summary of studies 1, 2, and 3. ADOHC: acute disease online health community; CDOHC: chronic disease online health community; COM: companionship support; PES: providing emotional support; PIS: providing information support; SES: seeking emotional support; SIS: seeking information support; UGC: user-generated content.

We present the main elements of the 3 studies in Figure 5, cascading from user profiling to social support and social network research. Combining these studies allows the conclusions to be mutually supportive of each other and also leads to new findings. Study 1 identified the overall distribution of user characteristics in the CDOHC and ADOHC, showing relevant attributes, such as disease, total UGC, etc, and clustering into different user groups based on these characteristics. Study 2 segmented user performance on social support in the CDOHC and ADOHC, and the statistical results reflected the differences in user needs for different social supports. Combining study 1 resulted in new findings. Study 3 focused on the exchange of post content between users and the characteristics of the various subnetworks they form. Studies 1-3 gradually uncovered the behaviors and needs of users and the connections between users in the community, and combining the microuser and macrocommunity perspectives made the conclusions richer and stronger.

## Discussion

With the rapid development of social media and digital health care, accelerated by the impact of the COVID-19 pandemic on traditional medical services, OHCs have become important channels for people to seek medical treatment and social support. These communities serve distinct purposes depending on the nature of the disease and shape how users engage, seek help, and express emotions. This study explored user behavior differences through comparative analysis of CDOHCs and ADOHCs, revealing how disease trajectories influence user behavior, support needs, and community dynamics.

CDOHCs encourage sustained engagement as users demonstrate increased activity levels and a dual requirement for both emotional and information support. This is consistent with the biopsychosocial model of illness, which underscores the interplay among biological, psychological, and social factors in chronic conditions. Using k-means clustering, we identified three key user profiles in the CDOHC: (1) newly diagnosed patients (mainly with diabetes or cancer) who exhibit low interaction levels and predominantly negative emotions, (2) transitioning patients who engage moderately but still express significant emotional struggle, and (3) long-term members who are highly active, deeply involved in the community, and generally positive in emotions. This pattern is consistent with the posttraumatic growth theory, which posits that individuals develop resilience after prolonged health challenges. Network analysis further revealed that medical terms in CDOHC clusters strongly linked disease terminology with emotional expressions, highlighting how chronic illness narratives intertwine clinical and psychosocial concerns.

In contrast, ADOHCs primarily function as transactional centers for urgent medical guidance, with interactions predominantly focused on informational assistance rather than emotional engagement and where users perceive acute conditions as time-sensitive but resolvable, reducing the need for prolonged emotional disclosure. There were two distinct subgroups in the ADOHC: (1) young patients with appendicitis showing minimal online participation due to urgent care needs and (2) older patients with cerebral hemorrhage displaying limited engagement associated with caregiving proxies or digital literacy barriers. Unlike the CDOHC, the ADOHC exhibited sparse emotional connections but concentrated on predominantly informational hubs, treatment efficacy, and time-sensitive treatment advice.

These findings have 3 significant theoretical implications. First, by constructing a disease-specific characteristic index system for user profiling in OHCs, this study provides a theoretical framework for the precise identification and differentiated understanding of patient users in OHCs. Through an analysis of variations in social support expressions across communities of different disease types, the heterogeneous needs of user groups at various health stages were revealed. Second, this research constructed a multilevel social support network based on user interaction patterns and developed an interaction behavior analysis framework tailored to the health context. The study further examined the density and structural heterogeneity of community support networks across different disease types, offering theoretical insights into user self-organization mechanisms and support strategies under diverse health predicaments. Third, this work advances the integration and interdisciplinary development of the social support theory, user profiling theory, and social network theory within health informatics.

The study findings also have several key practical implications. First, given the heightened emotional support needs of chronic disease users, key actions include implementing lightweight NLP analysis modules in forum backends to enable real-time mental health monitoring, integrating these NLP-based risk detection systems with clinician alert protocols for high-risk cases, and developing embedded interactive learning modules to cultivate peer support networks among trained volunteers. For the critical reliance of patients with acute diseases on rapid information access, platform administrators should implement AI-driven content verification systems to ensure information authenticity and validity of online health platforms and should strictly prevent the release and circulation of erroneous treatment information. All community users should contribute to platform quality by practicing responsible, evidence-based content standards and participating in gamified educational modules that promote constructive engagement. At the systemic level, these technical solutions should inform policy making, with governments supporting NLP tools for mental health screening and health care providers bridging online risk detection with offline interventions. Furthermore, strategic partnerships between digital platforms and community organizations can create hybrid support networks that combine the scalability of AI-enabled online services with the personalization of local volunteer programs, ultimately fostering a supportive patient care ecosystem.

## Abbreviations

ADOHC: acute disease online health community
CDOHC: chronic disease online health community
LDA: latent Dirichlet allocation
NLP: natural language processing
OHC: online health community
UGC: user-generated content
WCSS: within-cluster sum of squares
WHO: World Health Organization

## Data Availability

The datasets used or analyzed in this study can be obtained from the corresponding author upon reasonable request.
